# Dr. Thomas Herring Burchard

**Published:** 1896-12

**Authors:** 


### Obituary.

Dr. Thomas Herring Burchard, of New York, aged 48 years, died suddenly of cardiac disease, November 15, 1896, at his home, 7 East 48th street. He graduated from Bellevue Hospital Medical College in 1872, and is reported to have advised operative interference as early as 1873 in perforative perityphlitis (appendicitis). He was an active professional man, gifted as a writer and speaker as well as a teacher. He made numerous contributions to the literature of medicine, was a member of the leading medical societies and a surgeon to Charity Hospital, now known as New York City Hospital. Of late years he had established himself in summer practice at Saratoga. He had just returned from a tour abroad for the benefit of his health and was about to resume practice when his fatal illness overtook him.

